# Assessment of Eravacycline Antimicrobial Susceptibility in China During the First Year Following Regulatory Approval (2023–2024): A Real-World Study

**DOI:** 10.3390/microorganisms14010044

**Published:** 2025-12-24

**Authors:** Qiaolian Yi, Yi Li, Menglan Zhou, Ran Jing, Minya Lu, Yingchun Xu

**Affiliations:** Department of Laboratory Medicine, Peking Union Medical College Hospital, Chinese Academy of Medical Sciences and Peking Union Medical College, Beijing 100730, China

**Keywords:** antimicrobial susceptibility, clinical breakpoints, *Acinetobacter baumannii*, *Klebsiella pneumonia*, eravacycline

## Abstract

Eravacycline, a novel fluorocycline antimicrobial, was approved by China’s National Medical Products Administration (NMPA) in March 2023; however, clinical breakpoints and real-world data on its use in China remain limited. We conducted a retrospective, questionnaire-based analysis of eravacycline use across 21 provinces in China during the first year after NMPA approval (September 2023–September 2024). Data from 3369 patients who received eravacycline were collected. We analyzed the distribution of pathogens and specimens, reported in vitro susceptibility to eravacycline, imipenem, meropenem, tigecycline, and polymyxins and evaluated microbiological outcomes. *Acinetobacter baumannii* (52.0%, 1259/2419) and *Klebsiella pneumoniae* (26.1%, 631/2419) were the most commonly reported pathogens. High levels of carbapenem resistance were observed: 704 of 771 (91.3%) for *A. baumannii* and 323 of 392 (82.4%) for *K. pneumoniae*. In contrast, susceptibility to eravacycline was 95.5% (737/772) and 92.5% (297/321), respectively. Microbiological outcomes suggested potential activity against these resistant isolates, though post-treatment culture data were limited. This study demonstrates that eravacycline exhibited potent in vitro activity against prevalent carbapenem-resistant Gram-negative pathogens in real-world Chinese clinical practice during its first-year post-approval. Continuous monitoring of eravacycline resistance trends, together with prospective studies that correlate microbiological and clinical outcomes in specific infection types, will be crucial for defining its long-term therapeutic utility and the risk of resistance emergence.

## 1. Introduction

Multidrug-resistant Gram-negative pathogens remain a major global health threat [1,2]. Eravacycline (ERV), approved by the U.S. Food and Drug Administration (FDA) in 2018 for the treatment of complicated intra-abdominal infections in adults [3], demonstrates potent in vitro activity against main key Gram-negative organisms, with susceptibility rates typically >90% (2013–2017 data) [4]. Expert consensus groups, including the European Society of Clinical Microbiology and Infectious Diseases (ESCMID) and the Infectious Diseases Society of America (IDSA), have proposed ERV as a potential option for select carbapenem-resistant infections, particularly those caused by *Acinetobacter baumannii* and carbapenem-resistant Enterobacterales (CRE) [5,6]. However, its use in empirical therapy remains uncertain due to limited clinical efficacy data and pharmacokinetic considerations.

ERV was approved by China’s National Medical Products Administration (NMPA; formerly the China Food and Drug Administration, CFDA) in March 2023, but real-world clinical data remain limited. To address this, a nationwide project was launched to assess ERV’s therapeutic effectiveness during its first post-approval year.

Accurate antimicrobial susceptibility testing (AST) is essential for ERV’s rational use. However, breakpoints vary between the FDA and the European Committee on Antimicrobial Susceptibility Testing (EUCAST) [7,8], and clinical breakpoints for key pathogens are lacking in China. In response, ChinaCAST—the National Health Commission’s expert committee on Antimicrobial Susceptibility Testing and Standard Research of China—has established China-specific clinical breakpoints: for *A. baumannii* and *Klebsiella pneumoniae*, susceptibility is defined as a minimum inhibitory concentration (MIC) ≤ 1 mg/L (broth microdilution) or disk diffusion zone ≥ 15 mm [9].

As part of this nationwide project, we conducted a retrospective, questionnaire-based analysis of ERV’s real-world use in China during its first-year post-approval, with the primary objective of characterizing the spectrum of pathogens treated, antimicrobial susceptibility profiles—including against carbapenem-resistant Enterobacterales and *A. baumannii*—and associated microbiological outcomes, using the newly established ChinaCAST clinical breakpoints for interpretation.

## 2. Materials and Methods

### 2.1. Study Population and Data Collection

This retrospective study was based on a standardized questionnaire completed by clinicians at participating hospitals across 21 provinces in China. The questionnaire captured real-world clinical data on adult patients (≥18 years) who received ERV as definitive therapy between September 2023 and September 2024.

Eligible cases included hospitalized patients treated with ERV for documented or suspected bacterial infections. For each patient, the treating physician or designated investigator provided information on demographics, underlying conditions, infection characteristics (site, pathogen if available), antimicrobial treatment details, and clinical outcomes assessed at 30 days after initiation of ERV therapy.

Of the 3369 submitted questionnaires, microbiological testing results were not reported for 767 (22.8%). Among the remaining 2602 patients with available microbiological data, 2373 (91.2%) had monomicrobial infections and 229 (8.8%) had polymicrobial infections. In this study, only patients with monomicrobial infections were included in the subsequent analyses.

### 2.2. Antimicrobial Susceptibility Testing

AST was performed locally at each participating center using routine clinical methods, including broth microdilution, automated systems, or gradient diffusion strips, and interpreted according to established standards. As this was a retrospective, questionnaire-based study, detailed laboratory protocols including specific AST platforms, standardization procedures, and quality control measures were not uniformly documented across sites.

For ERV, susceptibility results were interpreted using the clinical breakpoints established by ChinaCAST [9] (Table 1). For other antimicrobial agents, MIC interpretations followed CLSI M100 [10] guidelines, except for polymyxins and tigecycline, which were assessed using EUCAST breakpoints [8]. Notably, tigecycline susceptibility for *A. baumannii* was interpreted using the FDA breakpoints for Enterobacterales [11], as species-specific criteria for *A. baumannii* are not available.

In this study, carbapenem resistance was assessed only among isolates that underwent susceptibility testing for imipenem or meropenem; ertapenem and doripenem were not included in the analysis. Carbapenem resistance was defined as in vitro resistance to at least one of these two agents. Specifically, for Enterobacterales, resistance was defined as an MIC ≥4 mg/L for either imipenem or meropenem, whereas for *A. baumannii*, the resistance threshold was set at an MIC ≥8 mg/L for either agent.

To minimize bias from repeated sampling, only the first isolate of a given species per patient was included in the susceptibility analysis. Furthermore, the number of isolates tested for each antimicrobial agent varied by pathogen and infection site, reflecting real-world clinical testing practices.

### 2.3. Bacteriological Outcomes of Antimicrobials

Bacteriological outcomes were assessed based on clinician-reported data collected through a standardized questionnaire at the end of ERV therapy or during follow-up. The following categories were used: (1) eradication—the baseline pathogen(s) was no longer isolated, and no new pathogen was detected during treatment or on the first day after discontinuation;(2) presumed eradication—clinical improvement was observed, but post-treatment cultures were not obtained or were non-evaluable; (3) eradication with reinfection—the baseline pathogen(s) was cleared, but a new pathogen was isolated after treatment completion; (4) persistence—the baseline pathogen(s) remained detectable at end of therapy; and (5) presumed persistence—lack of clinical improvement without obtainable post-treatment culture material. Bacteriological success was defined as the composite of eradication, presumed eradication, and eradication with reinfection (categories 1–3). Bacteriological failure encompassed persistence and presumed persistence (categories 4–5).

### 2.4. Statistical Analysis

Categorical variables were summarized as frequencies and percentages, using % (n/m). Formal hypothesis testing was performed using the chi-square test or Fisher’s exact test, as appropriate. A *p*-value < 0.05 was considered statistically significant. Statistical analyses were performed and graphs were plotted using R (Version 4.2.1) (https://cran.r-project.org).

## 3. Results

### 3.1. Microbiological Characteristics

**Figure 1:**
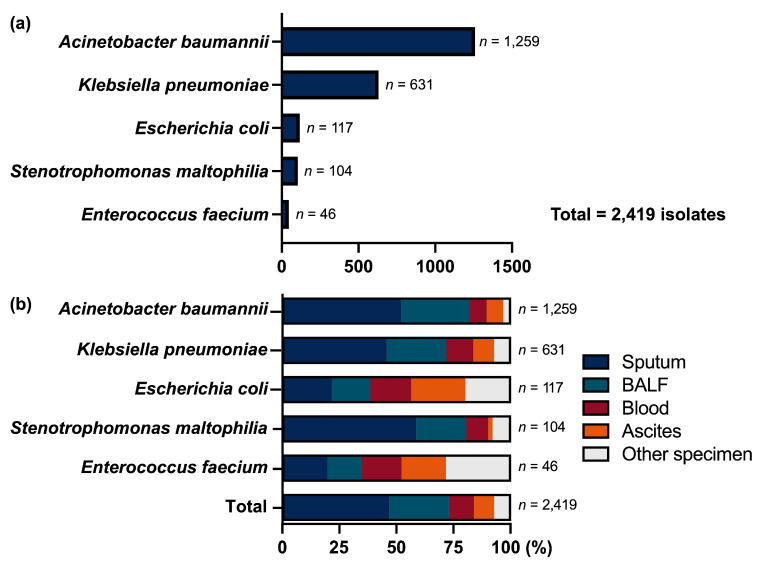
Pathogen distribution and specimen source among 2373 patients with monomicrobial infections. (**a**) Top 5 isolates pathogens (*n* = 2419 isolates). (**b**) Specimen source distribution by pathogen. BALF: bronchoalveolar lavage fluid.

Among 2373 cases with monomicrobial infections, a total of 2419 clinical specimens were submitted for microbiological analysis (Figure 1a). *A. baumannii* was the most frequently isolated pathogen (52.0%, *n* = 1259), followed by *K. pneumoniae* (26.1%, *n* = 631), *Escherichia coli* (4.8%, *n* = 117), *Stenotrophomonas maltophilia* (4.3%, *n* = 104), and *Enterococcus faecium* (1.9%, *n* = 46).

The specimen types associated with each pathogen reflected distinct infection sources (Figure 1b). Respiratory specimens predominated for *A. baumannii*, *K. pneumoniae*, and *S. maltophilia*: among these, sputum accounted for 51.9% (654/1259), 45.6% (288/631), and 58.7% (61/104) isolates, respectively, and bronchoalveolar lavage fluid (BALF) for 30.1% (379/1259), 26.3% (166/631), and 22.1% (23/104) isolates. In contrast, *E. coli* and *E. faecium* were primarily isolated from non-respiratory sites: *E. coli* (*n* = 117) was most commonly recovered from ascites (*n* = 28), sputum (*n* = 25), and blood (*n* = 21); *E. faecium* (*n* = 46) showed a similar pattern, with isolates from ascites (*n* = 9), sputum (*n* = 9), and blood (*n* = 8).

### 3.2. In Vitro Susceptibility Analysis of Eravacycline

ERV susceptibility data were available for 1515 bacterial isolates. After excluding duplicate isolates from the same patient, 1498 unique patient-derived isolates were analyzed using the clinical breakpoints established by ChinaCAST.

Among the major pathogens, the ERV susceptibility rates of *A. baumannii*, *K. pneumoniae*, *E. coli*, *E. faecium* and *S. aureus* were 95.5% (737/772), 92.5% (297/321), 72.1% (49/68), 41.7% (5/12), and 80.0% (4/5), respectively.

**Figure 2:**
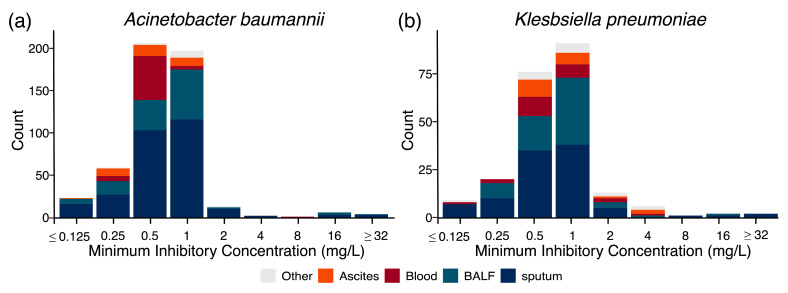
ERV MIC distribution by specimen type. (**a**) *A. baumannii*; (**b**) *K. pneumoniae*.

The MIC distributions of ERV against *A. baumannii* (*n* = 511) and *K. pneumoniae* (*n* = 220) were unimodal and approximately normal (Figure 2). ERV demonstrated potent in vitro activity against both species, with MIC_90_ values of 1 μg/mL. For *A. baumannii*, the MIC_50_, MIC_90_, and geometric mean (GM) were 0.5 μg/mL, 1 μg/mL, and 0.6404 μg/mL, respectively; for *K. pneumoniae*, the MIC_50_ and MIC_90_ were both 1 μg/mL, with a GM of 0.7253 μg/mL.

Overall, 94.9% (485/511) of *A. baumannii* and 89.1% (196/220) of *K. pneumoniae* isolates exhibited MICs ≤ 1 μg/mL—the susceptibility breakpoint defined by ChinaCAST for both species. This high rate of susceptibility was consistent across specimen types, including respiratory (sputum, BALF) and non-respiratory (blood, ascites) sources. Only a small number of isolates showed elevated MICs (2.2%, 11/511, for *A. baumannii*; 5/220, 2.3% for *K. pneumoniae*).

### 3.3. In Vitro Susceptibility Analysis of Carbapenems

**Figure 3:**
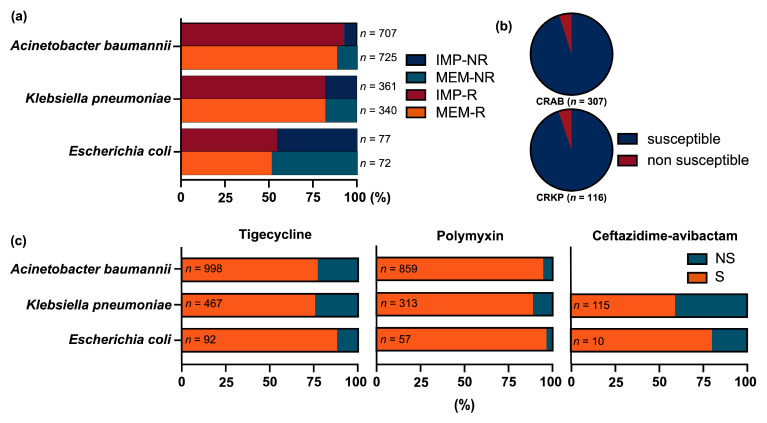
In vitro susceptibility of key Gram-negative pathogens to major antimicrobial agents. (**a**) Resistance rates to IMP and MEM by pathogen. (**b**) ERV susceptibility among carbapenem-resistant *A. baumannii* and *K. pneumoniae*. (**c**) Susceptibility to tigecycline, polymyxin, and ceftazidime–avibactam by pathogen. IMP, imipenem; MEM, meropenem; R: resistant; NR: non-resistant; CRAB: carbapenem-resistant *A. baumannii*; CRKP: carbapenem-resistant *K. pneumoniae*; S: susceptible; NS: non-susceptible.

Carbapenem susceptibility data were available for 1436 isolates tested against imipenem and 1449 isolates tested against meropenem. After excluding duplicate isolates from the same patient, 1410 and 1422 unique patient-derived isolates were analyzed for imipenem and meropenem, respectively (Figure 3a).

Among isolates tested for imipenem, resistance rates based on CLSI breakpoints were 92.8% (656/707) for *A. baumannii*, 82.0% (296/361) for *K. pneumoniae*, and 54.5% (42/77) for *E. coli*. Similarly, among meropenem-tested isolates, resistance rates were 88.4% (641/725), 82.1% (279/340), and 51.4% (37/72) for the same three species, respectively. Based on resistance to at least one carbapenem (imipenem or meropenem), 704 of 771 (91.3%) *A. baumannii* isolates were categorized as Carbapenem-resistant *A*. *baumannii* (CRAB), and 323 of 392 (82.4%) *K. pneumoniae* isolates as Carbapenem-resistant *K. pneumoniae* (CRKP).

The in vitro activity of ERV was assessed among *A. baumannii* and *K. pneumoniae* isolates with concomitant susceptibility results for both carbapenems (imipenem or meropenem) and ERV, interpreted using ChinaCAST breakpoints (Figure 3b). Among carbapenem-resistant isolates, 292 of 307 *A. baumannii* (95.1%) and 110 of 116 *K. pneumoniae* (94.8%) were susceptible to ERV. In contrast, all carbapenem-susceptible isolates—7 *A. baumannii* and 20 *K. pneumoniae*—were fully susceptible to ERV (100% susceptibility).

### 3.4. In Vitro Drug Susceptibility Analysis of Other Antimicrobials

In vitro susceptibility of key pathogens to major antimicrobial agents was also analyzed (Figure 3c). Tigecycline susceptibility was assessed in 1998 unique isolates (from 2033 submissions). When interpreted using EUCAST breakpoints—applicable only to *E. coli* and *S. aureus*—susceptibility rates were 39.1% (36/92) and 81.8% (9/11), respectively. Using FDA breakpoints (which include *A. baumannii* and *K. pneumoniae*), susceptibility rates were 77.2% (*A. baumannii*, 770/998), 75.6% (*K. pneumoniae*, 353/467), 88.0% (*E. coli*, 81/92), and 81.8% (*S. aureus*, 9/11).

Ceftazidime–avibactam data were available for 559 non-duplicate isolates (from 566 submissions); among these, 8 *E. coli* isolates showed 80.0% susceptibility, while 115 *K. pneumoniae* isolates exhibited 59.1% susceptibility per CLSI criteria. Finally, polymyxin susceptibility was evaluated in 1524 unique isolates (from 1556 submissions) using EUCAST breakpoints, yielding high susceptibility rates: 94.5% (812/859) for *A. baumannii*, 88.8% (278/313) for *K. pneumoniae*, and 96.5% (55/57) for *E. coli*.

### 3.5. In Vitro Susceptibility to Other Antimicrobials Among ERV-Susceptible A. baumannii and K. pneumoniae Isolates

Among ERV-susceptible isolates, notable variations of resistance to imipenem (IMP), meropenem (MEM), tigecycline (TGC), and polymyxin (POL) were observed across specimen types (Table 2). Data from ascites should be interpreted cautiously due to limited isolate numbers (*n* < 30), which may affect reliability and generalizability.

To improve clinical interpretability, we categorized specimens into two groups: sterile-site infections (blood and ascites) and respiratory samples (sputum and BALF), as surveillance or non-sterile site cultures (e.g., stool, throat swabs) were not included in this analysis.

For *A*. *baumannii*, among ERV-susceptible isolates, overall carbapenem resistance was high: 94.7% (392/414) to IMP and 91.0% (386/424) to MEM. In contrast, susceptibility to POL was high (95.8%, 520/543), while TGC showed moderate activity (78.4%, 495/631). Within respiratory specimens (sputum and BALF), IMP resistance ranged from 94.0% (BALF, 78/83) to 95.6% (sputum, 237/248), and MEM resistance from 87.8% (BALF, 79/90) to 92.2% (sputum, 235/255). POL susceptibility remained consistently high (94.2, 276/293, sputum; 99.3%, 149/150, BALF), and TGC susceptibility varied modestly (77.2%, 244/316, sputum; 79.3%, 146/184, BALF). Among sterile-site isolates, blood-derived strains exhibited IMP and MEM resistance rates of 92.5% (37/40) and 92.1% (35/38), respectively, with TGC and POL susceptibility at 80.3% (55/66) and 93.5% (43/46). In ascites, all isolates were resistant to both carbapenems (*n* = 6); however, all were susceptible to TGC (*n* = 20) and 95.0% (19/20) to POL.

For *K*. *pneumoniae*, among ERV-susceptible isolates, overall IMP and MEM resistance rates were 86.0% (153/178) and 86.7% (143/165), respectively, with POL susceptibility at 88.1% (163/185) and TGC at 72.1% (168/233). In respiratory specimens, carbapenem resistance varied between 82.8% (72/87, sputum, MEM) and 95.3% (41/43, BALF, IMP), while POL susceptibility ranged from 87.0% (80/92, sputum) to 89.8% (44/49, BALF), and TGC from 67.2% (45/67, BALF) to 76.1% (83/109, sputum). Among sterile-site isolates, blood-derived strains showed lower IMP resistance (75.0%, 15/20) compared to MEM (87.5%, 14/16), with TGC and POL susceptibility at 73.1% (19/26) and 94.7% (18/19), respectively. In ascites (*n* = 6), all isolates were resistant to both carbapenems, and susceptibility to both POL and TGC was 66.7% (4/6).

### 3.6. Microbiological Efficacy by Pathogen and Antimicrobial Susceptibility

Microbiological efficacy at the end of treatment varied by pathogen, antimicrobial agent, and baseline susceptibility profile (Table 3).

For *A*. *baumannii*, the overall microbiological success rate (defined as eradication plus presumed eradication) was consistently high regardless of carbapenem susceptibility. Among IMP-resistant isolates (*n* = 656), success was 90.1% (eradication: 41.6%; presumed eradication: 38.1%), compared with 91.9% for IMP-susceptible isolates (*n* = 37; eradication: 29.7%; presumed eradication: 45.9%). Similarly, MEM-resistant isolates (*n* = 641) achieved a 90.2% success rate (41.5% eradication; 38.5% presumed eradication), while MEM-susceptible isolates (*n* = 51) showed a slightly higher success rate of 94.1% (33.3% eradication; 45.1% presumed eradication). Notably, failure rates were low across all subgroups (5.9–9.9%).

In contrast, for *K*. *pneumoniae*, carbapenem resistance was associated with modestly improved microbiological outcomes. IMP-resistant isolates (*n* = 296) had an 88.9% success rate (43.6% eradication; 39.2% presumed eradication), whereas IMP-susceptible isolates (*n* = 59) showed a lower success rate of 84.7% (32.2% eradication; 42.4% presumed eradication), with a corresponding increase in failure (15.3% vs. 11.1%). A similar pattern was observed for meropenem: MEM-resistant isolates (*n* = 279) achieved 90.3% success (43.0% eradication; 40.5% presumed eradication), compared to 84.0% for MEM-susceptible isolates (*n* = 50, 28.0% eradication; 48.0% presumed eradication), with failure rates of 9.7% and 16.0%, respectively.

With respect to ERV, high microbiological success was observed irrespective of in vitro susceptibility classification. For *A. baumannii*, the overall success rate was 96.2% among ERV non-susceptible isolates (*n* = 26, 57.7% eradication; 26.9% presumed eradication) and 94.4% among ERV-susceptible isolates (*n* = 478, 43.5% eradication; 43.9% presumed eradication), with failure rates of only 3.8% and 5.6%, respectively. Similarly, for *K. pneumoniae*, success rates were 90.5% in the ERV non-susceptible group (*n* = 21, 47.6% eradication; 28.6% presumed eradication) and 94.4% in the ERV-susceptible group (*n* = 197, 50.3% eradication; 42.6% presumed eradication), with failure rates of 9.5% and 5.6%.

## 4. Discussion

Carbapenem resistance among Gram-negative pathogens remains a serious challenge [12,13]. In China, the prevalence of carbapenem-resistant *K. pneumoniae* (CRKP) has risen from approximately 3% to 22–25%, while carbapenem-resistant *A. baumannii* (CRAB) now exceeds 70% nationally—and reaches up to 90% in some centers [14]. CRAB-associated healthcare-associated infections are linked to mortality rates of 8–40% [15,16], yet therapeutic options remain severely limited [17], primarily restricted to tigecycline, polymyxins, and ceftazidime–avibactam.

Eravacycline, recently approved by the NMPA, is increasingly used for treatment of carbapenem-resistant organism (CRO) infections [18,19]. In our nationwide investigation, 91.3% (467/511) of *A. baumannii* and 82.4% (181/220) of *K. pneumoniae* isolates were carbapenem-resistant (based on imipenem or meropenem susceptibility). Despite this high baseline resistance, ERV demonstrated low clinical resistance rates and favorable in vitro activity: susceptibility rates were 95.1% (292/307) for CRAB and 94.8% (110/116) for CRKP, based on ChinaCAST MIC breakpoints tailored to the Chinese population.

Our data confirm that ERV retains potent activity against the two most challenging CROs in China. In contrast, tigecycline (TGC), historically used as a component of combination regimens for CRO [5], shows declining in vitro activity in contemporary surveillance [20,21]. In our study, resistance rates approached 30–40% among both CRAB and CRKP. While current guidelines conditionally support the use of high-dose TGC only when in vitro susceptibility is confirmed (e.g., for sulbactam-resistant CRAB when polymyxins are not preferred), its utility is increasingly constrained by rising MICs, variable tissue penetration, and lack of robust clinical outcome data [5]. Otherwise, patients enrolled in hospital-acquired pneumonia trials, particularly those with ventilator associated pneumonia and baseline bacteraemia, were at substantially increased risk of clinical failure and death when treated with tigecycline [22]. Consequently, agents with more consistent in vitro activity, such as ERV, may offer a microbiologically favorable alternative in settings where TGC susceptibility cannot be reliably assumed.

Polymyxins (colistin/polymyxin B), while often active in vitro, are associated with substantial safety concerns [23,24]. Large cohort studies and meta-analyses have consistently reported high rates of nephrotoxicity [25], ranging from 30% to over 50% [26], particularly when polymyxins are administered at higher loading doses, used in combination with other nephrotoxic agents (e.g., vancomycin, aminoglycosides), or given to patients with pre-existing renal impairment. In addition to dose-related toxicity, polymyxins suffer from poor lung penetration and emerging heteroresistance [27,28,29], further limiting their utility as monotherapy for serious CRO infections. Ceftazidime-avibactam, though highly effective against many KPC-producing Enterobacterales [30], has no activity against metallo-*β*-lactamases (e.g., NDM) or *A. baumannii*, rendering it ineffective against the majority of CRAB and a substantial proportion of CRKP in China, where *bla*_NDM_ dominates [31].

ERV exhibits potent in vitro activity against CRE, with MIC_90_ ≤ 0.5 mg/L and susceptibility rates >95%, even among *bla*_KPC_- and *bla*_NDM_-producing isolates [32,33,34,35]. No widespread acquired resistance mechanisms have been identified in Enterobacterales; although plasmid-mediated *tet*(X) variants confer high-level tetracycline resistance, they remain rare in clinical isolates [36,37]. In contrast, resistance in *A. baumannii* is primarily due to intrinsic efflux mechanisms (e.g., *tet*(B)) or, less commonly, acquired *tet*(X) variants, with prevalence of <3% [38,39].

Nevertheless, emerging threats warrant vigilance: *tet*(X3)–(X7) variants, detected mainly in animal sources and occasionally in human Enterobacterales in China, pose a risk of horizontal spread [36,37]. Expanded clinical use may select for *tet*(X)-positive or efflux-upregulated subpopulations [32,38], and heteroresistance in CRAB could lead to undetected resistance and treatment failure [39,40]. Ongoing surveillance of MIC trends, *tet*(X) determinants, and heteroresistance is essential to preserve ERV’s utility against CROs.

Our study extends these results to real-world clinical settings across China, a region characterized by exceptionally high antimicrobial resistance pressure, and confirms that ERV retains robust activity even under such challenging conditions. No ERV resistance was observed among carbapenem-resistant strains, underscoring its reliability across susceptibility strata.

One unexpected observation in our analysis was that patients infected with in vitro ERV-non-susceptible isolates appeared to have higher bacteriological eradication rates than those with susceptible strains for *A. baumannii*. However, this difference was not statistically significant, further supporting that it reflects methodological limitations rather than true biological efficacy. Specifically, (1) post-treatment cultures were rarely obtained, particularly in critically ill patients, and bacteriological outcomes were often inferred from clinical notes; (2) many patients with non-susceptible isolates received combination therapy (e.g., ERV plus carbapenems or polymyxins), which may have driven eradication; and (3) the small number of evaluable cases in the non-susceptible subgroup (*n* = 26 versus *n* = 478 in the susceptible group) increases vulnerability to random variation. Additionally, given ERV’s favorable tissue penetration, particularly in lung and intra-abdominal sites [41,42], local drug concentrations may exceed the MIC at the infection site, potentially enabling suppression despite systemic classification as non-susceptible.

There are several limitations to this study. First, as a retrospective observational analysis, unmeasured confounders, including infection severity (e.g., APACHE II or SOFA scores), comorbidities, source control, and concomitant therapies, were not adjusted for, precluding causal inference regarding any association between ERV susceptibility and outcomes. Second, data were collected via clinician-completed questionnaires without centralized adjudication, limiting assessment of inter-rater reliability. Third, antimicrobial regimens were not linked to individual MIC values or pharmacokinetic exposures; thus, our findings reflect microbiological trends rather than therapeutic efficacy. Finally, the absence of molecular characterization of resistance mechanisms (e.g., *bla*_OXA_, *bla*_NDM_, *bla*_KPC_) prevents genotype-phenotype correlation analyses.

Thus, while our data provide insights into ERV’s in vitro activity against CROs in a high-resistance setting, they should not inform clinical decision-making without confirmation from prospective, well-controlled studies with rigorous risk adjustment.

## 5. Conclusions

In summary, this retrospective study represents the nationwide baseline assessment of eravacycline antimicrobial susceptibility in China during the first year following its approval by the NMPA. Our findings support the appropriateness of the recently established ChinaCAST MIC breakpoints for ERV and underscore its potential as a valuable agent for the management of CRO infection. Future research should focus on long-term outcomes and resistance development to optimize ERV use. Longitudinal surveillance combining phenotypic susceptibility, MIC distributions, and carbapenemase genotyping will be essential to determine whether certain resistance determinants impact eravacycline activity or serve as early markers of emerging resistance.

## Figures and Tables

**Table 1:** The breakpoint (S/R) of ChinaCAST, EUCAST and FDA’s inhibitory zone diameter and MIC susceptibility tests for Eravacycline.

Organisms	Zone Diameter, Nearest Whole mm			MIC, μg/mL		
	ChinaCAST ^1^	EUCAST ^2^	FDA ^3^	ChinaCAST	EUCAST	FDA
*E. coli*	≥17/-	≥17/-	≥15/-	≤0.5/-	≤0.5/>0.5	≤0.5/-
*K. pneumoniae*	≥15/-	-/-	-/-	≤1/-	-/-	-/-
*A. baumannii*	≥15/-	-/-	-/-	≤1/-	-/-	-/-
*S. aureus*	≥20/-	≥20/<20	-/-	≤0.25/-	≤0.25/>0.25	≤0.06/-
*E. faecalis*	-/-	≥22/<22	-/-	≤0.125/-	≤0.25/>0.25	≤0.06/-
*E. faecium*	-/-	≥22/<22	-/-	≤0.125/-	≤0.25/>0.25	≤0.06/-

^1^ In 2024, ChinaCAST Clinical Antimicrobial Susceptibility Testing breakpoints. No resistant isolates are currently identified; hence, no resistance breakpoint is set. ^2^ In January 2024, EUCAST breakpoints for interpreting MICs and zone diameters, version 14.0. ^3^ In June 2022, FDA interpretive criteria for antimicrobial susceptibility testing. No resistant isolates are currently identified; hence no resistance breakpoint is set.

**Table 2:** Antimicrobial susceptibility distribution of eravacycline-susceptible *Acinetobacter baumannii* and *Klebsiella pneumoniae* among different specimens.

Pathogen	Specimen	IMP ^1^-Resistant (n/m, %)	MEM-Resistant (n/m, %)	TGC-Susceptible ^2^ (n/m, %)	POL-Susceptible (n/m, %)
*A. baumannii*	Overall	392/414, 94.7%	386/424, 91%	495/631, 78.4%	520/543, 95.8%
	Sputum	237/248, 95.6%	235/255, 92.2%	244/316, 77.2%	276/293, 94.2%
	BALF	78/83, 94%	79/90, 87.8%	146/184, 79.3%	149/140, 99.3%
	Blood	37/40, 92.5%	35/38, 92.1%	53/66, 80.3%	43/46, 93.5%
	Ascites	6/6, 100%	6/6, 100%	20/20, 100%	19/20, 95%
*K. pneumoniae*	Overall	153/178, 86%	143/165, 86.7%	168/233, 72.1%	163/185, 88.1%
	Sputum	77/92, 83.7%	72/87, 82.8%	83/109, 76.1%	80/92, 87%
	BALF	41/43, 95.3%	40/42, 95.2%	45/67, 67.2%	44/49, 89.8%
	Blood	15/20, 75%	14/16, 87.5%	19/26, 73.1%	18/19, 94.7%
	Ascites	5/5, 100%	6/6, 100%	4/6, 66.7%	4/6, 66.7%

^1^ IMP: Imipenem; MEM: Meropenem; TGC: Tigecycline; POL: Polymyxin. (n/m, %): among ERV-susceptible isolates with concomitant ANTIBIOTIC susceptibility testing, [n] of [m] ([X]%) were [resistant/susceptible] to ANTIBIOTIC. ^2^ FDA’s Breakpoints.

**Table 3:** Bacteriological outcomes of different antimicrobial groups (monomicrobial infection).

Pathogen	Group	Counts	Eradication	Presumed Eradication	Total Success	Failure	*p*-Value
*A. baumannii*	IMP-R	656	41.6%	38.1%	90.1%	9.9%	0.6158
	IMP-S	37	29.7%	45.9%	91.9%	8.1%	
	MEM-R	641	41.5%	38.5%	90.2%	9.8%	0.5909
	MEM-S	51	33.3%	45.1%	94.1%	5.9%	
	ERV-NS	26	57.7%	26.9%	96.2%	3.8%	0.3899
	ERV-S	478	43.5%	43.9%	94.4%	5.6%	
*K. pneumoniae*	IMP-R	296	43.6%	39.2%	88.9%	11.1%	0.4785
	IMP-S	59	32.2%	42.4%	84.7%	15.3%	
	MEM-R	279	43%	40.5%	90.3%	9.7%	0.1842
	MEM-S	50	28%	48%	84%	16%	
	ERV-NS	21	47.6%	28.6%	90.5%	9.5%	0.6424
	ERV-S	197	50.3%	42.6%	94.4%	5.6%	

IMP: Imipenem; MEM: Meropenem; R: resistant; S: susceptible; NS: non-susceptible.

## Data Availability

The data are not publicly available due to privacy or ethical restrictions.

## Abbreviations

The following abbreviations are used in this manuscript:

BALF	Bronchoalveolar lavage fluid
ChinaCAST	Committee of the National Health Commission on Antimicrobial Susceptibility Testing and Standard Research of China
CLSI	Clinical and Laboratory Standards Institute
CRAB	Carbapenem-resistant *Acinetobacter baumannii*
CRE	Carbapenem-resistant Enterobacteriaceae
CRKP	Carbapenem-resistant *Klebsiella pneumonia*
ERV	Eravacycline
ESCMID	European Society of Clinical Microbiology and Infectious Diseases
EUCAST	European Committee on Antimicrobial Susceptibility Testing
FDA	the US Food and Drug Administration
IDSA	Infectious Diseases Society of America
IMP	Imipenem
MEM	Meropenem
MIC	Minimum Inhibitory Concentration
NMPA	China’s National Medical Products Administration
POL	Polymyxin
TGC	Tigecycline

## References

[1] (2024). WHO Bacterial Priority Pathogens List, 2024: Bacterial Pathogens of Public Health Importance to Guide Research, Development and Strategies to Prevent and Control Antimicrobial Resistance.

[2] Macesic N., Uhlemann A.-C., Peleg A.Y. (2025). Multidrug-Resistant Gram-Negative Bacterial Infections. Lancet.

[3] Alosaimy S., Abdul-Mutakabbir J.C., Kebriaei R., Jorgensen S.C.J., Rybak M.J. (2020). Evaluation of Eravacycline: A Novel Fluorocycline. Pharmacother. J. Hum. Pharmacol. Drug Ther..

[4] Morrissey I., Olesky M., Hawser S., Lob S.H., Karlowsky J.A., Corey G.R., Bassetti M., Fyfe C. (2020). In Vitro Activity of Eravacycline against Gram-Negative Bacilli Isolated in Clinical Laboratories Worldwide from 2013 to 2017. Antimicrob. Agents Chemother..

[5] Paul M., Carrara E., Retamar P., Tängdén T., Bitterman R., Bonomo R.A., de Waele J., Daikos G.L., Akova M., Harbarth S. (2022). European Society of Clinical Microbiology and Infectious Diseases (ESCMID) Guidelines for the Treatment of Infections Caused by Multidrug-Resistant Gram-Negative Bacilli (Endorsed by European Society of Intensive Care Medicine). Clin. Microbiol. Infect..

[6] Tamma P.D., Aitken S.L., Bonomo R.A., Mathers A.J., van Duin D., Clancy C.J. (2022). Infectious Diseases Society of America 2022 Guidance on the Treatment of Extended-Spectrum β-Lactamase Producing Enterobacterales (ESBL-E), Carbapenem-Resistant Enterobacterales (CRE), and *Pseudomonas aeruginosa* with Difficult-to-Treat Resistance (DTR-*P. aeruginosa*). Clin. Infect. Dis..

[7] https://www.fda.gov/drugs/development-resources/eravacycline-injection-products.

[8] The European Committee on Antimicrobial Susceptibility Testing (2025). Breakpoint Tables for Interpretation of MICs and Zone Diameters. Version 15.0. https://www.eucast.org/fileadmin/eucast/pdf/breakpoints/v_15.0_Breakpoint_Tables.pdf.

[9] (2025). Expert Committee of the National Health Commission on Antimicrobial Susceptibility Testing and Standard Research; Clinical Microbiology Laboratory Specialized Committee of Chinese Hospital Association; Chinese Committee on Antimicrobial Susceptibility Testing. Specifications for Antimicrobial Susceptibility Testing of Eravacycline (2025). Chin. J. Lab. Med..

[10] Clinical and Laboratory Standards Institute (2025). Performance Standards for Antimicrobial Susceptibility Testing.

[11] https://www.fda.gov/drugs/development-resources/tigecycline-injection-products.

[12] Lee J., Sunny S., Nazarian E., Fornek M., Abdallah M., Episcopia B., Rowlinson M.C., Quale J. (2023). Carbapenem-Resistant Klebsiella Pneumoniae in Large Public Acute-Care Healthcare System, New York, NY, USA, 2016–2022. Emerg. Infect. Dis..

[13] Lei T., Liao B., Yang L.-R., Wang Y., Chen X. (2024). Hypervirulent and Carbapenem-Resistant Klebsiella Pneumoniae: A Global Public Health Threat. Microbiol. Res..

[14] Qin X., Ding L., Hao M., Li P., Hu F., Wang M. (2024). Antimicrobial Resistance of Clinical Bacterial Isolates in China: Current Status and Trends. JAC Antimicrob. Resist..

[15] Manjusha M., Yasasve M., Saravanan M., Belete M.A. (2023). Carbapenem-Resistant Acinetobacter Baumannii Healthcare-Associated Infections: Antimicrobial Resistance and Its Spread as a Global Threat. Int. J. Surg..

[16] Müller C., Reuter S., Wille J., Xanthopoulou K., Stefanik D., Grundmann H., Higgins P.G., Seifert H. (2023). A Global View on Carbapenem-Resistant *Acinetobacter baumannii*. mBio.

[17] Pogue J.M., Mann T., Barber K.E., Kaye K.S. (2013). Carbapenem-Resistant *Acinetobacter baumannii*: Epidemiology, Surveillance and Management. Expert. Rev. Anti Infect. Ther..

[18] Doi Y. (2019). Treatment Options for Carbapenem-Resistant Gram-Negative Bacterial Infections. Clin. Infect. Dis..

[19] Bartal C., Rolston K.V.I., Nesher L. (2022). Carbapenem-Resistant Acinetobacter Baumannii: Colonization, Infection and Current Treatment Options. Infect. Dis. Ther..

[20] Wang J.L., Lai C.C., Ko W.C., Hsueh P.R. (2023). Geographical Patterns of in Vitro Susceptibilities to Tigecycline and Colistin among Worldwide Isolates of *Acinetobacter baumannii*, Escherichia Coli and Klebsiella Pneumoniae: Data from the Antimicrobial Testing Leadership and Surveillance (ATLAS) Programme, 2016–2021. Int. J. Antimicrob. Agents.

[21] Wu Y., Chen J., Zhang G., Li J., Wang T., Kang W., Zhang J., Sun H., Liu Y., Xu Y. (2024). In-Vitro Activities of Essential Antimicrobial Agents Including Aztreonam/Avibactam, Eravacycline, Colistin and Other Comparators against Carbapenem-Resistant Bacteria with Different Carbapenemase Genes: A Multi-Centre Study in China, 2021. Int. J. Antimicrob. Agents.

[22] McGovern P.C., Wible M., El-Tahtawy A., Biswas P., Meyer R.D. (2013). All-Cause Mortality Imbalance in the Tigecycline Phase 3 and 4 Clinical Trials. Int. J. Antimicrob. Agents.

[23] Peng D., Zhang F., Chen Y., Zhao C., Niu J., Yang J., Li Z., Chen C., Qiu S., Zhang H. (2023). Efficacy and Safety of Colistin Sulfate in the Treatment of Infections Caused by Carbapenem-Resistant Organisms: A Multicenter Retrospective Cohort Study. J. Thorac. Dis..

[24] El-Sayed Ahmed M.A.E.-G., Zhong L.-L., Shen C., Yang Y., Doi Y., Tian G.-B. (2020). Colistin and Its Role in the Era of Antibiotic Resistance: An Extended Review (2000–2019). Emerg. Microbes Infect..

[25] Kaye K.S., Shorr A.F., Wunderink R.G., Du B., Poirier G.E., Rana K., Miller A., Lewis D., O’Donnell J., Chen L. (2023). Efficacy and Safety of Sulbactam–Durlobactam versus Colistin for the Treatment of Patients with Serious Infections Caused by Acinetobacter Baumannii–Calcoaceticus Complex: A Multicentre, Randomised, Active-Controlled, Phase 3, Non-Inferiority Clinical Trial (ATTACK). Lancet Infect. Dis..

[26] Nang S.C., Azad M.A.K., Velkov T., Zhou Q.T., Li J. (2021). Rescuing the Last-Line Polymyxins: Achievements and Challenges. Pharmacol. Rev..

[27] Jo J., Kwon K.T., Ko K.S. (2023). Multiple Heteroresistance to Tigecycline and Colistin in Acinetobacter Baumannii Isolates and Its Implications for Combined Antibiotic Treatment. J. Biomed. Sci..

[28] Roch M., Sierra R., Andrey D.O. (2023). Antibiotic Heteroresistance in ESKAPE Pathogens, from Bench to Bedside. Clin. Microbiol. Infect..

[29] Band V.I., Satola S.W., Smith R.D., Hufnagel D.A., Bower C., Conley A.B., Rishishwar L., Dale S.E., Hardy D.J., Vargas R.L. (2021). Colistin Heteroresistance Is Largely Undetected among Carbapenem-Resistant *Enterobacterales* in the United States. mBio.

[30] Shirley M. (2018). Ceftazidime-Avibactam: A Review in the Treatment of Serious Gram-Negative Bacterial Infections. Drugs.

[31] Zhang F., Liu X., Li Z., Li Z., Lei Z., Fan Y., Yang X., Liu Q., Ma Y., Lu B. (2025). Tracking International and Regional Dissemination of the KPC/NDM Co-Producing Klebsiella pneumoniae. Nat. Commun..

[32] Huang P.-Y., Hsu C.-K., Tang H.-J., Lai C.-C. (2024). Eravacycline: A Comprehensive Review of in Vitro Activity, Clinical Efficacy, and Real-World Applications. Expert. Rev. Anti Infect. Ther..

[33] Lee Y.-L., Ko W.-C., Lee W.-S., Lu P.-L., Chen Y.-H., Cheng S.-H., Lu M.-C., Lin C.-Y., Wu T.-S., Yen M.-Y. (2021). In-Vitro Activity of Cefiderocol, Cefepime/Zidebactam, Cefepime/Enmetazobactam, Omadacycline, Eravacycline and Other Comparative Agents against Carbapenem-Nonsusceptible Enterobacterales: Results from the Surveillance of Multicenter Antimicrobial Resistance in Taiwan (SMART) in 2017–2020. Int. J. Antimicrob. Agents.

[34] Galani I., Papoutsaki V., Karaiskos I., Moustakas N., Galani L., Maraki S., Mavromanolaki V.E., Legga O., Fountoulis K., Platsouka E.D. (2023). In Vitro Activities of Omadacycline, Eravacycline, Cefiderocol, Apramycin, and Comparator Antibiotics against *Acinetobacter baumannii* Causing Bloodstream Infections in Greece, 2020–2021: A Multicenter Study. Eur. J. Clin. Microbiol. Infect. Dis..

[35] Teo J.Q.-M., Chang H.Y., Tan S.H., Tang C.Y., Ong R.T.-H., Ko K.K.K., Chung S.J., Tan T.T., Kwa A.L.-H. (2023). Comparative Activities of Novel Therapeutic Agents against Molecularly Characterized Clinical Carbapenem-Resistant *Enterobacterales* Isolates. Microbiol. Spectr..

[36] Maraki S., Mavromanolaki V.E., Magkafouraki E., Moraitis P., Stafylaki D., Kasimati A., Scoulica E. (2022). Epidemiology and in Vitro Activity of Ceftazidime–Avibactam, Meropenem–Vaborbactam, Imipenem–Relebactam, Eravacycline, Plazomicin, and Comparators against Greek Carbapenemase-Producing *Klebsiella pneumoniae* Isolates. Infection.

[37] Ray S., Flemming L.K., Scudder C.J., Ly M.A., Porterfield H.S., Smith R.D., Clark A.E., Johnson J.K., Das S. (2025). Comparative Phenotypic and Genotypic Antimicrobial Susceptibility Surveillance in *Achromobacter* spp. through Whole Genome Sequencing. Microbiol. Spectr..

[38] Jean S.-S., Ko W.-C., Lu M.-C., Lee W.-S., Hsueh P.-R. (2022). Multicenter Surveillance of in Vitro Activities of Cefepime-Zidebactam, Cefepime-Enmetazobactam, Omadacycline, Eravacycline, and Comparator Antibiotics against *Enterobacterales*, *Pseudomonas aeruginosa*, and *Acinetobacter baumannii* Complex Causing Bloodstream Infection in Taiwan, 2020. Expert Rev. Anti-Infect. Ther..

[39] Li X., Gao P., Chen M., Li B., Xu X. (2025). In Vitro Activity of Eravacycline against Carbapenem-Resistant Gram-Negative Bacilli and Associated Risk Factors for Non-Susceptible Infections from a Tertiary Hospital in Fujian, China from 2021 to 2024. BMC Microbiol..

[40] Li Y., Chen X., Guo Y., Lin S., Wang M., Xu J., Wang X., He G., Tan X., Zhuo C. (2024). Emergence of Eravacycline Heteroresistance in Carbapenem-Resistant *Acinetobacter baumannii* Isolates in China. Front. Cell. Infect. Microbiol..

[41] Zhanel G.G., Cheung D., Adam H., Zelenitsky S., Golden A., Schweizer F., Gorityala B., Lagacé-Wiens P.R.S., Walkty A., Gin A.S. (2016). Review of Eravacycline, a Novel Fluorocycline Antibacterial Agent. Drugs.

[42] Connors K.P., Housman S.T., Pope J.S., Russomanno J., Salerno E., Shore E., Redican S., Nicolau D.P. (2014). Phase I, Open-Label, Safety and Pharmacokinetic Study To Assess Bronchopulmonary Disposition of Intravenous Eravacycline in Healthy Men and Women. Antimicrob. Agents Chemother..

